# Cardiovascular Risk Assessment and Lipid-Lowering Therapy Recommendations in Primary Prevention

**DOI:** 10.3390/jcm14072220

**Published:** 2025-03-25

**Authors:** Aikaterini Komnianou, Konstantinos G. Kyriakoulis, Ariadni Menti, Evangelos Dimakakos, George S. Stergiou, Anastasios Kollias

**Affiliations:** Hypertension Center STRIDE-7, School of Medicine, National and Kapodistrian University of Athens, Third Department of Medicine, Sotiria Hospital, 152 Mesogion Avenue, Athens 11527, Greece; katiakomn95@gmail.com (A.K.); konkyriakoulis@gmail.com (K.G.K.); ariamenti@yahoo.gr (A.M.); edimakakos@yahoo.gr (E.D.); stergiougs@gmail.com (G.S.S.)

**Keywords:** cardiovascular disease, primary cardiovascular prevention, lipid-lowering therapy, risk tools, statin, SCORE2, PCE, PREVENT

## Abstract

Cardiovascular disease remains the leading cause of morbidity and mortality worldwide, underscoring the importance of effective primary prevention strategies. Current total cardiovascular disease (CVD) risk assessment tools, such as the Systematic Coronary Risk Evaluation 2 (SCORE2) in Europe and the Pooled cohort equations (PCEs) and Predicting Risk of CVD EVENTs (PREVENT) in the USA, aim to identify individuals at high CVD risk and guide clinical decision-making in the primary prevention setting. Statin therapy reduces cardiovascular events and is recommended as the first step for individuals with estimated CVD risk above specific thresholds. Moreover, the presence of risk modifiers, as well as the detection of asymptomatic atherosclerosis, reclassifies low-moderate CVD risk individuals into higher risk categories, contributing to tailored therapeutic decisions in primary prevention. However, differences in the performance of the available CVD risk assessment tools, the recommended thresholds for intervention, and the treatment targets by scientific societies introduce considerable inconsistency to the statin therapy practices. In addition, physicians’ inertia and poor patients’ adherence contribute to inadequate dyslipidemia control rates. This narrative review examines the available evidence on the current most used CVD risk assessment tools and the respective lipid-lowering recommendations, and highlights the role of targeted screening for asymptomatic atherosclerosis in terms of individualized therapy for primary prevention.

## 1. Introduction

Cardiovascular disease (CVD) is the leading cause of morbidity and mortality worldwide [1,2,3]. The estimated number of CVD-related deaths in 2022 was around 19.8 million, most of which occurred in low- and middle-income countries [1]. These figures highlight the lack of effective primary CVD prevention strategies attributed to several factors such as suboptimal screening, difficulties in guidelines implementation and physicians’ inertia, as well as suboptimal patients’ drug adherence. Indeed, blood pressure and low-density lipoprotein (LDL) cholesterol control rates are rather disappointing [4,5].

Patients with established CVD are by definition at very high risk, and modifiable risk factors should be treated aggressively in the context of a secondary prevention strategy [1,2]. However, for individuals without established CVD, guidelines for CVD prevention recommend assessment of risk factors and of total CVD risk, and subsequent individualized pharmacological interventions [1,2]. In this context, relevant guidelines recommend the use of risk assessment models (risk scores) that consider several traditional risk factors to estimate the risk for future CVD events in individuals without established CVD (primary prevention) and identify those who will benefit from early treatment and control of the modifiable risk factors [1].

There are several available scores for CVD risk prediction proposed by the current guidelines in the primary prevention setting. In Europe, the 2021 European Society of Cardiology (ESC) guidelines on CVD prevention in clinical practice recommended the use of the Systematic Coronary Risk Evaluation 2 (SCORE2) for individuals aged 40–69 years [2]. Dedicated scores are also recommended for selected patient groups such as SCORE2-Older Persons (SCORE2-OP) for patients aged ≥70 years and SCORE2-Diabetes for patients with diabetes mellitus (DM) [2,6,7]. The American College of Cardiology (ACC)/American Heart Association (AHA) recommended the use of the Pooled cohort equations (PCEs) in the 2019 AHA/ACC Guidelines [8]. Recently, an updated risk score was published on behalf of the AHA, namely the Predicting Risk of CVD EVENTs (PREVENT) [9]. PREVENT claims to more accurately predict CVD risk in a large, diverse, and contemporary sample of USA adults by using routinely available clinical variables but also by including additional variables such as the estimated glomerular filtration rate (eGFR), the body mass index (BMI), and the use of statin therapy [9].

The aim of this narrative review is to provide a general overview of the available CVD risk scores endorsed by the current guidelines in Europe and the USA in the primary prevention setting, to discuss the role of potential risk modifiers and asymptomatic atherosclerosis detection in the risk reclassification, and to summarize current recommendations regarding lipid-lowering therapy.

## 2. CVD Risk Calculation Tools

### 2.1. SCORE2

In 2003, the ESC recommended the use of SCORE as a 10-year risk prediction algorithm for fatal CVD outcomes [10,11]; however, certain issues restricted its longevity. First, SCORE was developed from cohorts before 1986, rendering it questionable for contemporary European populations. Second, the populations in the different European regions present heterogeneity in their baseline CVD risk, and a single equation for all regions may not accurately estimate their risk. Third, SCORE predicts only fatal CVD outcomes and therefore underestimates the overall CVD risk. To address these limitations, the ESC recently developed and incorporated SCORE2, which is an updated version of SCORE [12].

SCORE2 estimates the 10-year risk for fatal total CVD and non-fatal atherosclerotic CVD outcomes and is derived from 45 prospective cohort studies (13 European countries) with 677,684 individuals aged 40–69 years, with the baseline survey conducted between 1990 and 2009 and the median follow-up at 10.7 years [12]. Fatal CVD outcomes included death due to coronary heart disease, heart failure, arrhythmias, stroke, abdominal aortic aneurysms, and sudden death, and non-fatal CVD outcomes included non-fatal myocardial infarction and non-fatal stroke [12]. This equation has been externally validated in 1,133,181 individuals from 15 European countries [12]. Since patients with similar risk factors in different regions appear to present different total CVD risks, SCORE2 was calibrated to four risk category regions (low, moderate, high, and very high CVD risk) based on national CVD mortality rates reported in a World Health Organization (WHO) study in 2021 [2].

SCORE2 is intended for use in individuals aged 40–69 years without established CVD or diabetes, and its use is recommended by the ESC guidelines [2]. The SCORE2 algorithm has not been validated and consequently cannot be used in patients with familial hypercholesterolemia or other genetic lipid disorders, as well as in patients with secondary hypertension [2]. Parameters used in the SCORE2 algorithm include sex, age, current smoking, systolic blood pressure (SBP), total cholesterol and high-density lipoprotein (HDL) cholesterol (Table 1).

Older individuals represent a special category at high CVD risk and thus a different score is needed to offer optimal risk predictions [7]. The SCORE2-OP project involved 28,503 individuals aged ≥65 years without pre-existing CVD who were recruited during the period 1994–2003 in Norway with a median follow-up of 13 years [7]. The primary outcome included fatal total CVD and non-fatal atherosclerotic CVD events. For the external validation, 338,615 individuals were analyzed from six study populations [7]. The recalibrated risk-adjusted SCORE2-OP estimates the 10-year CVD risk in persons aged ≥70 years and is based on the same parameters as SCORE2 [7].

DM is a major risk factor for CVD [6]. In previous guidelines, patients with DM were homogeneously considered as high CVD risk [6]; however, patients with DM represent a heterogeneous group based on various parameters such as the disease onset and the long-term control (time in range). To clarify this issue, the SCORE2-Diabetes algorithm has been developed, which estimates the 10-year CVD risk for fatal total CVD and non-fatal atherosclerotic CVD outcomes in patients with DM without established CVD [6]. Data were derived from a sample of 229,460 individuals aged ≥40 years with DM and without previous established CVD from four different datasets, assessed between 2006 and 2017. The median follow-up varied from 6.0 to 11.3 years depending on the dataset involved. The external validation was performed in 217,036 patients in four different countries [6]. SCORE2-Diabetes has also been recalibrated in four risk regions. The SCORE2-Diabetes algorithm incorporates the use of three additional parameters compared to SCORE2: the age of diabetes onset, the recent HbA1c, and the recent eGFR [6].

### 2.2. PCE

PCE estimates the 10-year risk of a first atherosclerotic CVD event and is derived from four prospective cohort studies in the USA with 24,626 individuals aged 20–79 years, with the baseline survey conducted between 1984 and 1993 and the follow-up period for over 12 years [1,13]. Atherosclerotic CVD events included coronary heart disease death, non-fatal myocardial infarction, and fatal or non-fatal stroke. The external validation was performed in 3785 individuals [1,13]. This score is better applied to non-Hispanic Whites and Blacks living in the USA [8].

PCE is intended for use in individuals aged 40–75 years without established CVD and is recommended in the 2019 ACC/AHA guidelines for primary CVD prevention [8]. This score has not been validated and consequently cannot be used in patients with familial hypercholesterolemia or other genetic lipid disorders [13]. Parameters used in the PCE score include age, sex, race, total cholesterol, HDL-cholesterol, SBP, antihypertensive treatment, diabetes diagnosis, and smoking status (Table 1) [1].

### 2.3. PREVENT

PREVENT estimates the 10- and 30-year risk of fatal and non-fatal total (atherosclerotic and heart failure) CVD events and is derived from 25 prospective cohort and electronic medical record datasets in the USA with 3,281,919 individuals aged 30–79 years, with the baseline survey conducted between 1992 and 2017 and the mean follow-up at 4.8 years [9]. External validation was performed in 3,330,085 participants from 21 additional datasets [9].

PREVENT is intended for use in individuals aged 30–79 years without established CVD [9]. In 2023, the AHA Cardiovascular-Kidney-Metabolic Scientific Advisory Group proposed PREVENT as a calibrated algorithm for estimating the 10-year CVD risk [9]. PREVENT has separate calculators for estimating risk of heart failure, atherosclerotic CVD risk, and total CVD (atherosclerotic plus heart failure) risk [14]. Moreover, PREVENT assigns lower risk estimates for atherosclerotic CVD events versus PCE and this difference increases with age and in the Black race [9]. Parameters for calculation of the PREVENT score include age, sex, BMI, eGFR, total cholesterol, HDL-cholesterol, SBP, antihypertensive and statin treatment, diabetes diagnosis, and smoking status (Table 1) [9]. The expanded model of the PREVENT score has some additional variables such as HbA1c, urine albumin-creatinine ratio, and social deprivation index [9].

### 2.4. Limitations of Risk Scores

Although CVD risk scores represent evidence-based equations with acceptable performance, their use is influenced by some limitations. First, these provide predictions only at a certain statistical and population level with an ‘acceptable’ margin of error. Second, they do not take into consideration a variety of factors such as the diastolic blood pressure levels, the dynamic blood pressure variation, the existence of hypertension phenotypes such as white-coat or masked hypertension, the inconsistency in the cardioprotective effects of very high levels of HDL-cholesterol, the inability to incorporate the duration of the previous exposure to a risk factor (i.e., a high smoking load, or a delayed initiation of antihypertensive and/or hypolipidemic treatment), the inability to distinguish whether normal values of a risk factor are due to current drug treatment, the presence of other important risk-modifiers that are not considered, such as the socioeconomic deprivation, the family history of premature CVD, the presence of auto-immune inflammatory diseases, and the lifestyle habits.

## 3. CVD Risk Classification per Risk Score

### 3.1. CVD Risk Classification—SCORE2

**Figure 1:**
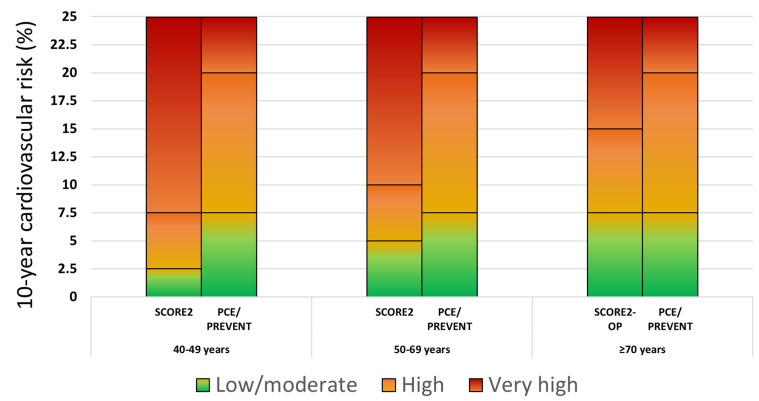
Cardiovascular disease risk categories based on the SCORE2, SCORE2-OP, PCE, and PREVENT scores. Footnote to Figure 1: For reasons of comparability, SCORE2 low-moderate, high and very high risk categories corresponded to the low and borderline, intermediate and high PCE/PREVENT risk categories, respectively.

According to SCORE2, individuals are assigned to one out of three risk categories (low-moderate, high, and very high) using risk thresholds depending on age (Figure 1).

Specifically, the risk thresholds for defining the low-moderate, high, and very high risk categories are as follows: for <50 years old, <2.5%, 2.5 to <7.5%, and ≥7.5%, respectively; for 50–69 years old, <5%, 5 to <10%, and ≥10%, respectively; for ≥70 years old, <7.5%, 7.5 to <15%, and ≥15%, respectively. For patients aged 40–69 years with DM, SCORE2-Diabetes classification is independent of age and includes the low risk (<5%), the moderate risk (5% to <10%), the high risk (10% to <20%) and the very high risk categories (≥20%) [15].

### 3.2. CVD Risk Classification—PCE Score

According to PCE, individuals are assigned to one out of four risk categories independent of age: the low risk (<5%), borderline risk (5% to <7.5%), intermediate risk (7.5% to <20%), and high risk (≥20%) (Figure 1). Individuals with DM aged 40–75 years belong to the intermediate or high risk category.

### 3.3. CVD Risk Classification—PREVENT Score

The classification according to PREVENT is currently exactly the same as with PCE (this is also the case with the expanded PREVENT score).

## 4. Risk Modifiers and the Role of Detecting Asymptomatic Atherosclerosis

Risk score algorithms provide rough estimates of the future CVD risk. Apart from the classic risk factors incorporated in these equations, there are various other factors that act as risk modifiers and might reclassify total CVD risk. Prompt detection of these factors will allow more accurate CVD risk estimation and more appropriate therapeutic decisions. Some examples of risk modifiers include family history of premature CVD, auto-immune inflammatory conditions (e.g., rheumatoid arthritis, systemic lupus erythematosus, or psoriasis), increased BMI, gestational hypertension, number of antihypertensive medications received, C-reactive protein, lipoprotein (a), N-terminal pro-B-type natriuretic peptide, eGFR, high sensitivity troponin-T, and indices of asymptomatic target-organ damage, i.e., increased coronary artery calcium score (CAC), presence of carotid plaque, albuminuria and abnormal ankle-brachial index [2,16]. Another important risk modifier is familial hypercholesterolemia, which is caused by functional mutations in the LDL receptor, apolipoprotein B, or Proprotein Convertase Subtilisin/Kexin type 9 (PCSK9) genes [2]. Individuals with familial hypercholesterolemia, even in the absence of other risk factors, are considered at least at high CVD risk. The Dutch Lipid Clinic Network diagnostic criteria are used to identify those with familial hypercholesterolemia and should be applied when there is a high suspicion of this diagnosis [2]. Moreover, several genetic variants have been linked to increased risk for coronary heart disease, and multilocus genetic risk scores have been developed to identify high risk individuals [17].

Among these risk modifiers, special reference should be made to the presence of asymptomatic atherosclerosis. The detection and quantification of subclinical atherosclerosis improves CVD risk prediction over classic risk scores alone and contributes to the reclassification of low-moderate CVD risk patients to the category of high or very high CVD risk [18,19,20]. With this respect, non-invasive imaging, including CAC, coronary computed tomography angiography, and carotid ultrasonography, as well as imaging linked to functional stress testing (stress echocardiography, cardiac magnetic resonance imaging, single photon emission computed tomography, or positron emission tomography), have an important role in accurately predicting CVD risk in asymptomatic patients [21,22]. Especially, CAC and carotid ultrasonography might be useful for selected but wide-ranging screening purposes. CAC is standardized, has solid evidence on its predictive ability, is strongly recommended by the AHA guidelines in cases of uncertain clinical decisions, but is associated with exposure to radiation and inability to detect noncalcified plaques [8,23]. Carotid ultrasonography also carries superior prognostic value versus available scores, is widely available and easy to perform, has the ability to detect noncalcified plaques, and does not involve radiation exposure; however, its accuracy is operator-dependent and there is a lack of a standardized assessment method and reporting [8,24,25]. Carotid atherosclerosis can be detected in a significant proportion of the general population above the age of 50 years, which would automatically translate to high CVD risk and statin eligibility [26]. Thus, it becomes clear that this examination might refine CVD risk prediction and ameliorate treatment decisions. Moreover, data show that carotid ultrasonography might increase the patients’ adherence to drug treatment [27]. The available evidence derived from outcome studies indicates that when ultrasonographic detection of atherosclerosis is implemented, low-moderate CVD risk individuals will be reclassified into a higher CVD risk category in significant percentages (Table 2). Lastly, it should be mentioned that asymptomatic peripheral arterial disease diagnosed through routine screening in the primary care setting by performing ankle-brachial index measurements has been shown to predict high mortality and/or vascular event risk [28].

## 5. Recommendations for Statin Administration in Primary CVD Prevention

All available guidelines state that individuals should adopt lifestyle changes to reduce their CVD risk, including smoking cessation, following healthy diets and engaging in at least 150 min of moderate-intensity activity or 75 min of vigorous-intensity activity per week [2,8]. In addition to these essential lifestyle changes, risk assessment tools help identify individuals who also require statin therapy [2,8]. Recommendations for initiating statin treatment based on the risk algorithm, as well as treatment targets used, are presented below.

### 5.1. 2021 ESC Guidelines

**Figure 2:**
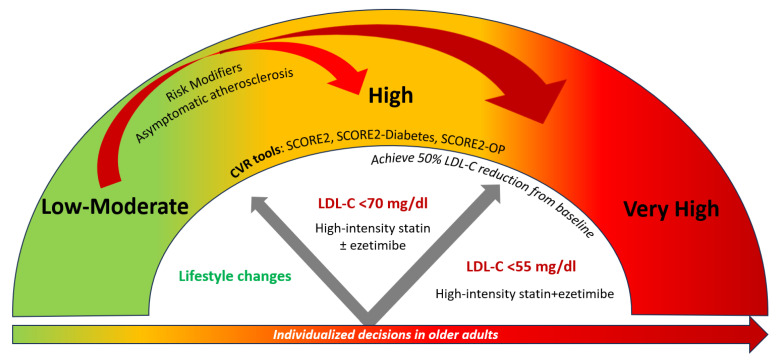
Treatment decisions and LDL-cholesterol targets depending on CVD risk classification according to SCORE2, SCORE2-Diabetes, and SCORE2-OP.

CVD risk categories do not ‘automatically’ translate into recommendations for starting drug treatment. On an individualized level, consideration of risk modifiers, lifetime CVD risk, treatment benefit and safety, comorbidities, frailty, and patient preferences may further guide treatment decisions [2]. Since age is a major driver of CVD risk, but lifelong risk factor treatment benefit is higher in younger people, the risk thresholds for considering treatment are lower for younger people [2]. Statin treatment is recommended for apparently healthy people who are at very high CVD risk (SCORE2 ≥ 7.5% for age under 50 years; SCORE2 ≥ 10% for age 50–69 years; SCORE2-OP ≥ 15% for age ≥ 70 years) and should be considered for those at high CVD risk (SCORE2 2.5 to <7.5% for age under 50 years; SCORE2 5 to <10% for age 50–69 years; SCORE2-OP 7.5 to <15% for age ≥ 70 years) [2]. For individuals at low-moderate risk (SCORE2 < 2.5% for age under 50 years; SCORE2 < 5% for age 50–69 years; SCORE2-OP < 7.5% for age ≥ 70 years), treatment is not generally recommended except in the presence of risk modifiers that reclassify the risk of a patient or if the expected treatment benefits are deemed significant [2]. A stepwise treatment intensification approach is recommended with ultimate LDL-cholesterol targets of <70 mg/dL for high CVD risk and <55 mg/dL for very high CVD risk individuals while achieving LDL-cholesterol reduction of ≥50% from baseline [2]. The latter practically translates to the use of the maximum dose of high-intensity statin if tolerated or the use of statin/ezetimibe combinations [29]. Similar decisions are taken for individuals with DM [2]. The suggested algorithm for treatment decisions and targets based on CVD risk assessment is presented in Figure 2. According to the ESC guidelines, CAC scoring or carotid plaque detection may be considered to improve CVD risk classification when the latter is initially around treatment decision thresholds [2].

### 5.2. 2019 ACC/AHA Guidelines

In individuals at borderline CVD risk (PCE 5% to <7.5%), the presence of risk-enhancing factors may justify initiation of moderate-intensity statin therapy [8]. In adults at intermediate CVD risk (≥7.5% to <20%), a moderate-intensity statin should be recommended, targeting reduction of LDL-cholesterol by 30% or more [8]. It should be noted that risk-enhancing factors favor intensification of statin therapy in the latter group. Statin therapy is recommended in individuals at high CVD risk (≥20%), targeting a reduction of LDL-cholesterol by 50% or more [8]. In adults with DM who have multiple CVD risk factors, it is reasonable to prescribe high-intensity statin therapy with the aim to reduce LDL-cholesterol levels by 50% or more [8].

In a 2022 report by the USA Preventive Services Task Force on the evidence of the benefits and harms of statins for reducing CVD-related morbidity or mortality, it was suggested that statins should be recommended for individuals aged 40–75 years with PCE CVD risk of ≥10% and on a selective basis for those with PCE CVD risk of 7.5 to <10% [30].

The recently developed PREVENT score has not been implemented in any recent ACC/AHA guidelines update.

### 5.3. Selecting Statin Therapy in Primary Prevention

**Figure 3:**
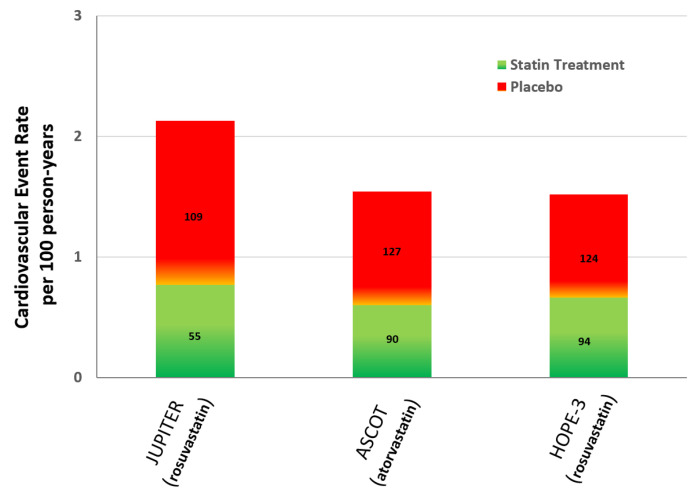
Cardiovascular event rate with statins versus placebo in randomized primary prevention trials involving more than 10,000 participants [34,35,36]. Footnote to Figure 3: Numbers within each column correspond to the achieved mean LDL-cholesterol levels (mg/dL).

Statin therapy is the first-line treatment for high cholesterol and CVD risk reduction. Several studies have confirmed their effectiveness in the primary and secondary CVD prevention setting [31,32,33]. Major randomized primary prevention trials involving more than 10,000 participants have shown that the CVD event rate reduction with statin use is dependent on the achieved LDL-cholesterol levels (Figure 3) [34,35,36].

Statins are classified into three categories based on their intensity: low-, moderate-, and high-intensity (Table 3) [32]. The intensity of a statin is determined by both the specific drug and its dosage. Low-intensity statins reduce LDL-cholesterol by <30%, moderate-intensity by 30% to <50%, and high-intensity by ≥50% [32]. The intensity of treatment should be determined by the severity of hypercholesterolemia, the CVD risk, and the LDL-cholesterol target [37]. Based on the European guidelines, a high-intensity statin at the highest tolerated dose should be selected to achieve LDL-cholesterol goals set for the high risk groups [2].

## 6. Other Hypolipidemic Drugs for LDL-Cholesterol Reduction and CVD Prevention

When the LDL-cholesterol target is not achieved with the maximum tolerated statin dose or in cases of statin intolerance, additional treatment strategies can be applied.

Ezetimibe has been shown to be effective in primary CVD prevention in older individuals, suggesting the importance of the LDL-cholesterol-lowering strategy [38]. Its use represents the second treatment step after titrating statin for achieving LDL-cholesterol target in high/very high CVD risk patients [2]. When high-intensity statin is combined with ezetimibe, LDL-cholesterol is reduced by approximately 65% [2]. In addition, strategies implementing lower doses of high potency statin combined with ezetimibe versus higher doses of high potency statin alone in very high CVD risk patients have been proved to be non-inferior with a higher proportion of patients achieving LDL-cholesterol target and lower intolerance-related drug discontinuation or dose reduction [39,40].

Bempedoic acid is a novel oral non-statin related drug that acts via a pathway reducing LDL-cholesterol but not predisposing to myalgia. A meta-analysis of randomized clinical trials confirmed that bempedoic acid significantly reduced LDL-cholesterol by 23% [41]. Most importantly, it has been shown to reduce major adverse CVD events among statin-intolerant patients [42]. It can be combined with ezetimibe in cases of statin intolerance for achieving LDL-cholesterol targets [2].

PCSK9 inhibitors represent an emerging new therapeutic option for the treatment of dyslipidemia [43,44]. Alirocumab and evolocumab are subcutaneously injectable monoclonal antibodies against PCSK9 administered every 2 or 4 weeks, approved for the treatment of hypercholesterolemia in very high CVD risk patients not achieving LDL-cholesterol targets with high-intensity statin and ezetimibe therapy or with intolerance to statins [2]. They can reduce LDL-cholesterol by ~60% as monotherapy [2], and when combined with a high-intensity statin or statin plus ezetimibe, LDL-cholesterol reduction reaches ~75% and ~85%, respectively [2]. Their efficacy in CVD prevention has been confirmed in the secondary prevention setting [45,46]. Inclisiran is subcutaneously administered once every 6 months and reduces PCSK9 levels through an RNA interference mechanism, leading to approximately a 50% reduction in LDL-cholesterol levels [47]. Preliminary results seem to be promising about its effectiveness in reducing CVD events [48].

Lastly, although LDL-cholesterol is the primary target and the treatment strategy includes drugs that aim to reduce it, recent evidence suggests that icosapent ethyl reduces CVD events in patients with established CVD or patients with diabetes and additional CVD risk factors who have high triglyceride levels and relatively well controlled LDL-cholesterol levels on statin therapy [49].

## 7. Implementation of Current Guidelines, Real-World Control Rates, and Perspectives

Several factors involving the healthcare system, physicians and patients themselves may contribute to achieving or not the LDL-cholesterol treatment goals. It is questionable if guideline recommendations are optimally implemented in real-world everyday clinical practice. In the Da Vinci study, which was conducted between 2017 and 2018, among 3000 primary and 2888 secondary CVD prevention treated patients, only 33% of patients achieved the LDL-cholesterol treatment goals [50]. The recent Santorini study, which was conducted between 2020 and 2021, included 9044 patients at high or very high CVD risk and demonstrated that only 21% of patients achieved LDL-cholesterol goals [5]. Results from the 1-year follow-up of the Santorini study showed that among patients not achieving treatment goals, only one third of the patients received an escalation in lipid-lowering therapy [51]. Monotherapy and combination therapy usage rose from 54% and 26% to 57% and 38%, respectively. Goal attainment improved from 21% to 31%, and it was greater with combination therapy compared with monotherapy at follow-up (39% vs. 26%) [51]. The large cross-sectional EUROASPIRE V study, which was conducted across 78 centers in 16 European countries, assessed the extent to which European guidelines on CVD prevention were accurately implemented [52]. Data from 2759 participants indicated poor risk factor control, with only 34% receiving treatment for dyslipidemia. Among those treated, more than half (53%) had an LDL-cholesterol level of ≥100 mg/dL [52].

The differences in the CVD risk estimation provided by the available algorithms and the heterogeneity in the current recommendations for statin therapy might insert confusion in primary care decisions and further contribute to the recorded poor control rates of CVD risk factors [53,54]. The recently developed PREVENT score appears to lead to considerably lower statin eligibility for primary prevention compared to PCE since it assigns lower 10-year atherosclerotic CVD risk estimates whereas risk classification thresholds remain the same [14,53]; yet, the available evidence shows that the majority of the adults eligible for receiving statin therapy based on PREVENT do not report statin use in real-world clinical practice [14]. This had led to an expression of concern, with several experts suggesting that until ACC/AHA guidelines are updated, the use of the 2013 PCE calculator “should remain standard practice” or the PREVENT score should be implemented but with a parallel lowering of the thresholds for statin therapy [53]. The opinion of this writing group is that this difference might enhance clinical inertia and further aggravate poor patients’ adherence to statin therapy and that routine arterial imaging in middle-aged individuals at low-moderate CVD risk might lead to more effective prevention strategies [23,24,25,54].

Patients with reported statin intolerance represent a challenging condition for clinicians. A recent individual participant data meta-analysis of large-scale, randomized, double-blind trials indicated that statin therapy during the first year produced only a 7% relative increase in muscle pain or weakness [55]. Interestingly, most of all reports of muscle symptoms by statin-treated participants were not due to the statin [55]. In addition, evidence suggests that the nocebo effect driven by access to online information may be contributing to statin intolerance [56]. Overestimating statin intolerance might lead to unsatisfactory lipid control rates. In a simulation study among 130,778 patients with hypercholesterolemia and high or very high CVD risk, 9% appeared with statin intolerance, and only 8% of these attained the LDL-cholesterol target [57]. Ezetimibe, bempedoic acid, as well as their combinations with low-dose (or every other day administration of statin) might be very helpful in achieving lipid targets in patients at high CVD risk and reported statin intolerance [39,40,42,58].

## 8. Conclusions

The most widely used tools for assessing CVD risk include SCORE2 in Europe and PCE, as well as its recent update, PREVENT, in the USA. These scores have been developed and validated using data from large populations and perform well in predicting CVD, at least at the general population level. Current guidelines recommend high-intensity statin therapy targeting LDL-cholesterol reduction ≥50% in individuals at very high CVD risk. ESC guidelines also set specific LDL-cholesterol treatment targets. Statin therapy is considered in individuals at high/very high CVD risk and on a selective basis in those at low-moderate risk. Risk modifiers, especially detection of atherosclerosis, might optimize accurate CVD risk assessment and should be considered on a selective basis in low-moderate CVD risk individuals.

## Figures and Tables

**Table 1:** Risk factors included in total cardiovascular risk calculators.

Variables	SCORE2	PCE	PREVENT
Age	✓	✓	✓
Sex	✓	✓	✓
Smoking	✓	✓	✓
Race		✓	
Diabetes	Special version	✓	✓
Total cholesterol	✓	✓	✓
HDL-cholesterol	✓	✓	✓
Systolic blood pressure	✓	✓	✓
Antihypertensive therapy		✓	✓
Statin therapy			✓
eGFR			✓
Body mass index			✓

eGFR, estimated glomerular filtration rate; HDL, high-density lipoprotein; PCE, Pooled cohort equation; PREVENT, Predicting Risk of cardiovascular disease EVENTs; SCORE2, Systematic Coronary Risk Evaluation2.

**Table 2:** Recent studies in primary prevention evaluating the role of ultrasonographic detection of atherosclerotic plaque in terms of cardiovascular risk reclassification.

Study	Country	N	Age (y)	Mean Age (years)	Males N (%)	Statin N (%)	NRI of Carotid Plaque Detection (%)	Score Used and Number of Low-Moderate CVD Risk Individuals N (%)	Participants Reclassified to Higher CVD Risk by Detecting Atherosclerotic Plaque N (%)
Fuster 2024 [18]	USA	5716	55–80	59	2475 (43)	1665 (29)	Not reported	Framingham Risk ≤ 10%, 3312 (58)	2360/3312 (71)
Bao 2023 [19]	Sweden	4588	46–68	57	1788 (39)	81 (2)	46.1	SCORE2 Risk < 2.5–5%, 1663 (36)	344/1663 (21)
Nicolaides 2022 [20]	Cyprus	985	40–84	58	448 (45)	141 (14)	16.6	Based on classic risk factors 10-year risk < 7.5%, 478 (49)	56/478 (12)

CVD, Cardiovascular Disease; NRI, Net Reclassification Improvement; SCORE2, Systemic Coronary Risk Estimation 2.

**Table 3:** Statin treatment intensity.

Low-Intensity	Moderate-Intensity	High-Intensity
Pravastatin 10–20 mg	Pravastatin 40, 80 mg	Atorvastatin 40–80 mg
Simvastatin 5–10 mg	Simvastatin 20, 40, 80 mg	Rosuvastatin 20–40 mg
	Pitavastatin 1–4 mg	
	Atorvastatin 10–20 mg	
	Rosuvastatin 5–10 mg	

## Abbreviations

The following abbreviations are used in this manuscript:

ACC	American College of Cardiology/AHA
AHA	American Heart Association
BMI	Body mass index
CAC	Coronary artery calcium score
CVD	Cardiovascular disease
DM	Diabetes mellitus
eGFR	Estimated glomerular filtration rate
ESC	European Society of Cardiology
HDL	High-density lipoprotein
LDL	Low-density lipoprotein
PCE	Pooled cohort equation
PCSK9	Proprotein Convertase Subtilisin/Kexin type 9
PREVENT	Predicting Risk of cardiovascular disease EVENTs
SBP	Systolic blood pressure
SCORE2	Systematic Coronary Risk Evaluation 2
SCORE2-OP	SCORE2-Older Persons
WHO	World Health Organization
